# Does Leader AI-Focused Attention Promote Employee Proactivity? A Work-Related Rumination Theory Perspective

**DOI:** 10.3390/bs16060987

**Published:** 2026-06-13

**Authors:** Lu Xiao, Heng Zhao, Jin Wan

**Affiliations:** 1 School of Economics and Management, Beijing Jiaotong University, Beijing 100044, China; 24120666@bjtu.edu.cn; 2 School of Economics and Management, East China Jiaotong University, Nanchang 330013, China; 2934@ecjtu.edu.cn

**Keywords:** leader AI-focused attention, problem-solving pondering, affective rumination, AI job role clarity, proactive behavior

## Abstract

With the increasing embeddedness of AI robots and other intelligent technologies in organizational workplaces, leader AI-focused attention has emerged as an important reference point for employees as they use and adapt to AI-related technologies. Drawing on work-related rumination theory, this study develops and tests an integrated mediation model to examine how leader AI-focused attention is related to employee proactive behavior through two parallel pathways: problem-solving pondering and affective rumination. It further investigates the moderating role of AI job role clarity. Based on structural equation modeling of multi-wave survey data from 514 employees, the results show that leader AI-focused attention positively predicts employees’ problem-solving pondering and affective rumination. Problem-solving pondering is positively related to employee proactive behavior, whereas affective rumination is negatively related to employee proactive behavior. In addition, AI job role clarity positively moderates the relationship between leader AI-focused attention and problem-solving pondering; specifically, this positive relationship is stronger when employees report higher AI job role clarity. From the perspective of work-related rumination, this study extends the explanation of the psychological mechanisms linking leader AI-focused attention to employee proactive behavior. It also provides theoretical insights and practical implications for understanding the boundary condition of leaders’ attentional signals in AI-related work contexts and for supporting employee proactive behavior.

## 1. Introduction

As artificial intelligence has evolved from an efficiency-enhancing tool into a core force driving strategic organizational change (Budhwar et al., 2023), its workplace applications have continued to expand. Some organizations have begun to deploy embodied AI systems in settings such as customer service, production operations, on-site guidance, and task assistance, including service robots, industrial robotic arms, and intelligent guidance devices. In this development, leaders, as interpreters of organizational strategy and key decision makers in resource allocation, provide an important reference point for employees’ use of AI. In particular, leaders’ attention to specific AI applications in daily management (Dong et al., 2025) constitutes an important external context through which employees perceive and respond to AI-related work situations. However, existing research has mainly focused on the application of AI technologies themselves or on employees’ direct human–AI interaction (Wu et al., 2024; Wu et al., 2025; Wu & Zhang, 2024). Less is known about how employees interpret and respond to leader AI-focused attention and how this attention is associated with their self-initiated and future-oriented work behaviors. Clarifying this top-down process is therefore important for understanding how organizational vitality and employee initiative may be fostered in the AI era.

A review of the existing literature suggests that research at the intersection of leadership and AI has mainly developed along three streams. One stream examines AI-enabled leadership, focusing on how leaders use AI tools for decision analysis, team management, and performance enhancement (Joshi, 2025; Mohammed et al., 2025; Florea & Croitoru, 2025). Another stream focuses on AI symbolism, namely how leaders communicate the importance of and support for AI technologies through their words and actions, thereby shaping employees’ readiness for organizational change and their evaluations of leadership effectiveness (Hu et al., 2025; He et al., 2023). In addition, emerging studies have begun to examine leaders’ supportive behaviors during AI implementation, such as providing training and clear communication, and their potential role in reducing employees’ anxiety and enhancing acceptance (Sha et al., 2025; H.-Y. Wang et al., 2025; Sarkar, 2025; Van Quaquebeke & Gerpott, 2024). These studies provide an important foundation for understanding the role of leaders in AI-related change. However, they tend to position employees as passive adapters or responders, paying insufficient attention to the deeper proactive psychological processes and behavioral motives that may emerge after employees perceive leaders’ intentions. As a core dimension of employees’ positive work behavior, employee proactive behavior refers to self-initiated and discretionary actions that go beyond formal role requirements and are intended to improve one’s work situation or the organizational environment. It includes proactive learning, problem solving, and process improvement and serves as an important basis for organizational adaptation and sustainable development (Grant & Ashford, 2008; Fay & Frese, 2001; Crant, 2000; Li et al., 2023). In the context of AI-driven changes in work paradigms, employee proactive behavior is no longer confined to traditional work domains. It is also reflected in employees’ active adaptation to AI technologies, their use of AI tools to improve work effectiveness, and their exploration of ways to integrate AI into work processes. This raises an important question: Is leaders’ high level of attention to AI interpreted by subordinates as a developmental signal that may encourage initiative, or is it perceived as a source of performance pressure and role threat that may be associated with avoidance and delay? The complex mechanism underlying this process warrants further investigation.

To unpack this black box, this study draws on work-related rumination theory as its core explanatory framework and examines employees’ psychological responses to leader AI-focused attention from the perspective of cognitive and affective processing. Work-related rumination refers to repeated thinking about work-related issues during nonwork time and is commonly classified into problem-solving pondering and affective rumination (Querstret & Cropley, 2012). Problem-solving pondering is a constructive and solution-oriented form of repetitive thinking, often accompanied by positive expectations and future-oriented planning. By contrast, affective rumination reflects a passive form of repetitive thinking in which attention is centered on negative emotions and worries. Leader AI-focused attention constitutes an important work-related stimulus for employees. On the one hand, it may be interpreted as encouragement for innovation and adaptation, thereby making employees more likely to engage in constructive thinking about the future possibilities of AI-enabled work. On the other hand, it may also be perceived as a signal of potential skill obsolescence and role replacement, thereby making employees more likely to experience anxiety and uncertainty.

The extent to which employees engage in the aforementioned rumination pathways, as well as the intensity of such engagement, may also be shaped by their cognitive frames of interpretation. Among these factors, AI job role clarity represents a key boundary condition. AI job role clarity refers to employees’ cognition, understanding, and expectations regarding their work design, role positioning, scope of responsibilities, and task requirements in an AI-enabled collaborative work environment (Chowdhury et al., 2022). When employees have a clear understanding of their role requirements in the AI context, leader AI-focused attention is more likely to be interpreted as a specific developmental guide, thereby prompting problem-solving pondering about the use of AI tools, the optimization of work processes, and the development of relevant capabilities. Conversely, when AI job roles are ambiguous, employees may find it difficult to discern the specific requirements conveyed by leader AI-focused attention. In this case, they are more likely to interpret such attention as an uncertain source of pressure and to engage in affective rumination about skill adaptation, role changes, and performance expectations. Accordingly, this study develops a moderated dual-mediation model to examine the differentiated pathways through which leader AI-focused attention is associated with employee proactive behavior via affective rumination and problem-solving pondering, as well as the moderating role of AI job role clarity in these pathways.

**Figure 1:**
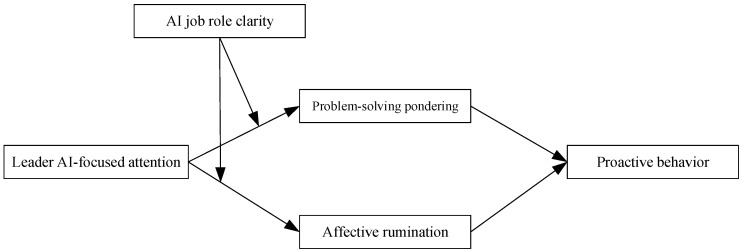
Theoretical model.

This study makes several theoretical contributions. First, from a research perspective, this study treats leader AI-focused attention as an important contextual signal and situates it within specific work settings involving AI robots. AI robots should not be viewed as entities detached from AI; rather, they represent a more concrete, observable, and interactive form through which AI technology is encountered in organizations. It is through these specific work settings that employees perceive leaders’ sustained attention to AI-related issues. By focusing on how leader AI-focused attention is associated with subordinates’ underlying behavioral motives, this study helps clarify how employees interpret and respond to leaders’ attentional signals in human–AI collaboration. Second, this study extends work-related rumination theory to AI-related work contexts by explaining how employees may continue to engage in cognitive and affective processing during nonwork time after perceiving leader AI-focused attention. This offers a useful theoretical lens for understanding employees’ nonwork-time psychological activities in AI-related work settings. Third, this study distinguishes between two mediating pathways, namely affective rumination and problem-solving pondering, which reflect affective and cognitive processing, respectively. It further introduces AI job role clarity as a cognitive moderator to explain how employees’ cognitive clarity may shape their affective and cognitive processing of work-related information during nonwork time. Overall, this study provides a more refined explanatory framework for understanding how leader AI-focused attention is associated with employee proactive behavior from an integrated cognitive and affective perspective. The theoretical model of this study is shown in Figure 1.

## 2. Theoretical Analysis and Research Hypotheses

### 2.1. Leader Al-Focused Attention

Leader AI-focused attention refers to leaders’ behavior of focusing their attentional resources on AI, reflected in employees’ perceptions of the extent to which their leaders continuously attend to specific AI applications, such as AI robots, in daily work. This concept emphasizes the direction of leaders’ attention toward specific AI applications and is distinct from digital transformation leadership and AI monitoring. Research on digital leadership focuses more on how leaders reshape communication patterns, organizational relationships, and change capabilities in digital technology environments (Bauwens & Cortellazzo, 2025). AI monitoring research treats technological systems as performance management tools, emphasizing how organizations use electronic systems to observe, record, analyze, and evaluate employees’ work activities, performance information, and related behavioral data, such as electronic performance monitoring (Ravid et al., 2023; Wolff et al., 2024; König, 2025). Dong et al. (2025) placed the allocation of leaders’ attentional resources in AI-related work contexts and suggested that leader AI-focused attention can serve as a social informational cue, leading employees to attach greater importance to AI-related information during AI talk and strengthening the process through which AI talk is associated with AI work crafting via AI self-efficacy. Thus, leader AI-focused attention is neither a broad form of digital leadership capability nor a form of technological monitoring of employees’ behavior or performance data. Rather, it represents a leadership attentional signal perceived by employees in specific AI application contexts, such as AI robots. Therefore, empirical research is needed to examine how and when leader AI-focused attention is associated with employee work outcomes.

### 2.2. The Double-Edged Effect of Leader Al-Focused Attention on Proactive Behavior

#### 2.2.1. The Mediating Role of Problem-Solving Pondering

Unlike AI leadership, which emphasizes leaders’ overall capability to drive AI-related change, and unlike technology-oriented leadership, which concerns how leaders promote managerial communication, organizational coordination, technological innovation, and digital transformation through advanced information technologies, digital tools, and information systems (Avolio et al., 2000; Van Wart et al., 2019; Tagscherer & Carbon, 2023), leader AI-focused attention focuses more specifically on whether leaders continuously direct their attention, in everyday work interactions, to the specific operational status and behavioral manifestations of AI, such as the battery level, movement trajectory, obstacles, or verbal behaviors of AI robots during interaction (Dong et al., 2025).

According to work-related rumination theory, repetitive thinking during nonwork time is typically triggered by unresolved or highly salient work-related stimuli encountered during the workday. These stimuli are cognitively encoded as unfinished events and continue to activate individuals’ cognitive systems after they have left the work context. When leaders frequently display this type of specific and sustained attention in daily work, employees may become aware that AI-related work involves a series of issues that require further response, including modes of human–AI collaboration, the handling of abnormal situations, the embedding of AI into work processes, and the adaptation of relevant capabilities. This perception may strengthen the sense of incompleteness associated with AI-related tasks in employees’ cognition, making them more likely to continue thinking about these issues after work. From the perspective of control theory, individuals tend to attend to discrepancies between their current state and their desired state and to engage in repeated thinking as a way to reduce such discrepancies (Martin & Tesser, 1996). Leaders’ sustained attention to AI-related operational information and behavioral manifestations may therefore lead employees to perceive a gap between their current AI collaboration capabilities, problem-handling abilities, or work methods and future work requirements, thereby prompting continued cognitive processing during nonwork time. Because the information indicated by this type of leader attention is relatively specific, employees’ repetitive thinking is more likely to shift toward practical coping strategies rather than remain at the level of general impressions about technology. For example, employees may repeatedly think about how to collaborate with AI, how to intervene when AI operates abnormally, or how to use AI tools to optimize their own work processes, thereby actively exploring and constructing action pathways at the cognitive level. Accordingly, the following hypothesis is proposed:

**Hypothesis** **1a.** *Leader AI-focused attention is positively related to problem-solving pondering.*

Employee proactive behavior refers to self-directed, future-oriented behavior through which individuals anticipate and actively change the current conditions of their work, team, or organization (Griffin et al., 2007). Problem-solving pondering, as a deep and constructive form of cognitive processing directed toward work tasks, problems, and potential solutions, can enhance individuals’ understanding of the current situation and their anticipation of future developments through structured reflection (Grant & Ashford, 2008). When employees engage in problem-solving pondering, they are more likely to develop forward-looking thinking and a willingness to initiate change (Aspinwall & Taylor, 1997). In addition, work characteristics that facilitate deep cognitive processing, such as the criticality of work events, can stimulate employee proactive behavior by enhancing work engagement (Zhang et al., 2022). This problem-solving-oriented form of pondering can strengthen individuals’ sense of control and self-efficacy, thereby providing the key psychological resources and “can-do” beliefs required for proactive action (Parker et al., 2010). Particularly in dynamic and uncertain work environments, problem-solving pondering helps employees identify opportunities for improvement, anticipate potential obstacles, and develop coping strategies. Such cognitive preparation constitutes an important prerequisite for initiating self-directed change-oriented behavior (Koen & Parker, 2020). Therefore, this study hypothesizes that the more strongly employees engage in problem-solving pondering, the more likely they are to display employee proactive behavior at work.

**Hypothesis** **1b.** *Problem-solving pondering is positively related to employee proactive behavior.*

Leader AI-focused attention, as a work-related signal with a clear AI-specific focus, directs employees’ attention to issues such as AI-related tasks, work process adjustments, and capability alignment, and may lead them to continue thinking about these issues during nonwork time. Such problem-solving pondering is not a mere repetition of work-related pressure; rather, it involves sustained cognitive processing of problem causes, potential opportunities, and feasible courses of action. As this cognitive processing unfolds, employees may develop a clearer understanding of the relationship between AI-related changes and their own work, and become more likely to form preliminary ideas about work improvement, human–AI collaboration, and process optimization. This cognitive preparation provides a foundation for subsequent self-initiated and future-oriented action, making employees more likely to improve the current conditions of their work, teams, or organizations through proactive adjustment, exploration, and improvement (Griffin et al., 2007). Accordingly, this study argues that a higher level of leader AI-focused attention may be indirectly and positively associated with employee proactive behavior through problem-solving pondering.

**Hypothesis** **1c.** *Leader AI-focused attention is indirectly and positively associated with employee proactive behavior through problem-solving pondering.*

#### 2.2.2. The Mediating Role of Affective Rumination

Affective rumination refers to a state in which individuals continue to think involuntarily and repeatedly about work related negative emotions and stressful experiences after leaving the work context and remain immersed in worry, frustration, or insecurity (Querstret & Cropley, 2012). In a context in which artificial intelligence is rapidly reshaping organizations, leader AI-focused attention significantly shapes employees’ perceptions and evaluations of the work environment. Based on work rumination theory, when individuals are confronted with threatening or uncertain work demands, persistent and difficult to suppress negative thinking tends to be activated in order to process highly aroused negative emotional states. When employees perceive that their leaders are highly focused on AI, while they themselves feel inadequately prepared in relevant skills or uncertain about the direction of change, substantial anxiety and insecurity are likely to be experienced (Teng et al., 2023). This continuing anxiety and threat perception triggered by external stressors (Feng, 2024) drives individuals to process, although often ineffectively, psychological discomfort that is difficult to resolve, and affective rumination is therefore likely to occur (Guo et al., 2025). Accordingly, the following hypothesis is proposed:

**Hypothesis** **2a.** *Leader AI-focused attention is positively related to affective rumination.*

Employee proactive behavior is an extra-role behavior through which individuals seek to improve themselves and their environment and actively initiate change. Such behavior typically requires sufficient cognitive resources and an action-oriented psychological state. However, affective rumination, as a repetitive and intrusive thinking process centered on negative work-related experiences, may continuously occupy and deplete limited cognitive resources, leaving individuals with insufficient psychological energy to plan future actions and pursue change (Sonnentag, 2018). This recurring cycle of negative thinking may intensify and prolong emotions such as anxiety and frustration, which may erode the psychological capital required to initiate and sustain employee proactive behavior (McCarthy et al., 2016). From a behavioral perspective, immersion in affective rumination may hinder the psychological detachment necessary for recovery and aggravate emotional exhaustion, which may strengthen the motivational tendency to maintain the status quo and avoid additional challenges (Sonnentag, 2003). As a result, employees’ willingness to proactively solve problems, improve work methods, or assume extra-role responsibilities may be weakened (Ohly & Fritz, 2010). Therefore, affective rumination is expected to be negatively related to employee proactive behavior.

**Hypothesis** **2b.** *Affective rumination is negatively related to employee proactive behavior.*

Although leader AI-focused attention can signal to employees that the organization values AI applications and technological adaptation, this signal does not necessarily translate into positive employee action. For some employees, leaders’ sustained attention to AI may intensify concerns about skill updating, role changes, and job replacement, leading AI-related requirements to be interpreted as work-related information imbued with pressure and threat. In such circumstances, employees are more likely to repeatedly think about whether their own capabilities are sufficient to cope with AI-induced changes and whether future work requirements will exceed their existing competence. Such affective rumination may keep individuals immersed in anxiety, uncertainty, and feelings of inadequacy, thereby reducing the likelihood that they will transform external requirements into action readiness. Because employee proactive behavior typically requires considerable psychological energy, willingness to solve problems, and future orientation, when employees primarily process leader AI-focused attention through affective rumination, their willingness to actively seek change, explore new methods, and solve problems may be inhibited. Accordingly, the following hypothesis is proposed.

**Hypothesis** **2c.** *Leader AI-focused attention is indirectly and negatively associated with employee proactive behavior through affective rumination.*

### 2.3. The Moderating Role of AI Job Role Clarity

AI job role clarity refers to the extent to which employees clearly understand their roles, responsibilities, and expected outcomes in the context of artificial intelligence application (Budhwar et al., 2023). Employees with higher AI job role clarity are better able to understand the specific work requirements and goals implied by leader AI-focused attention, and uncertainty and anxiety caused by role ambiguity may therefore be reduced (Nawaz et al., 2024). This clear role cognition makes it more likely that leader AI-focused attention will be interpreted as an opportunity to improve personal effectiveness and career development, and employees are thus more likely to engage actively in AI-related problem-solving pondering and to explore how AI tools can be used to optimize work processes and improve work efficiency (Xu et al., 2023). When AI job role clarity is low, by contrast, employees may feel confused because their specific roles in AI application are not clearly understood. As a result, positive responses to leader AI-focused attention may be weakened, and resistance may even emerge, making problem-solving pondering less likely. It can therefore be expected that AI job role clarity strengthens the positive relationship between leader AI-focused attention and problem-solving pondering. At the same time, because problem-solving pondering serves as a key antecedent of employee proactive behavior, this indirect relationship is also likely to become stronger when AI job role clarity is high. Accordingly, the following hypotheses are proposed:

**Hypothesis** **3.** *AI job role clarity positively moderates the relationship between leader AI-focused attention and problem-solving pondering.*

**Hypothesis** **4.** *AI job role clarity positively moderates the indirect relationship between leader AI-focused attention and employee proactive behavior through problem-solving pondering.*

When AI job role clarity is high, employees are able to clearly understand their responsibilities and expectations in AI-related tasks, thereby reducing uncertainty and anxiety arising from role ambiguity (Sonnentag, 2003). Such clear role cognition helps employees interpret leader AI-focused attention as a developmental opportunity rather than a threat, thereby reducing the frequency and intensity of affective rumination. By contrast, when AI job roles are unclear, employees may feel confused because they cannot accurately understand task requirements. As a result, when confronted with leader AI-focused attention, they are more likely to fall into affective rumination, which may in turn undermine employee proactive behavior. Accordingly, the following hypotheses are proposed:

**Hypothesis** **5.** *AI job role clarity negatively moderates the relationship between leader AI-focused attention and affective rumination.*

**Hypothesis** **6.** *AI job role clarity negatively moderates the indirect relationship between leader AI-focused attention and employee proactive behavior through affective rumination.*

## 3. Method

### 3.1. Research Procedure and Sample

The data were collected from current employees of technology firms in China who had experience collaborating or interacting with AI robots in their daily work. The survey was conducted between November 2025 and January 2026 through Credamo, a professional online survey platform. Credamo has established respondent-screening procedures and data-quality control mechanisms, and its participant pool includes working adults from a wide range of industries. The platform has also been used in empirical research in management and psychology (Yu et al., 2023; Z. Wang & Jiang, 2023). Prior to formal data collection, this study received approval from the Science and Technology Ethics Committee of Beijing Jiaotong University.

Before the main questionnaire, two screening questions were used to confirm whether respondents met the eligibility criteria for the study. The first question assessed the type of organization: “Does your company operate in a technology-related industry, such as artificial intelligence, information technology, high-end equipment manufacturing, or software development?” The response options were: (1) “Yes” and (2) “No.” The second question assessed respondents’ AI-related work experience: “In your daily work, are you directly or indirectly involved in collaboration or interaction with AI robots, such as service robots, industrial robotic arms, or intelligent guidance devices?” The response options were: (1) “Yes, frequently”; (2) “Yes, occasionally”; and (3) “No, rarely or almost never.” Only respondents who answered “Yes” to the first question and selected option (1) or (2) for the second question were invited to continue with the survey. After this screening procedure, 614 eligible employees were recruited for the study.

To reduce the potential for common method bias, this study adopted a three-wave survey design, with a four-week interval between adjacent waves. In the first wave, data on leader AI-focused attention, AI job role clarity, and participants’ demographic information were collected. A total of 587 valid responses were obtained, yielding a valid response rate of 95.60%. In the second wave, participants who had completed the first wave were invited to continue the survey, and data on affective rumination and problem-solving pondering were collected. A total of 552 valid responses were obtained, yielding a valid response rate of 94.04%. In the third wave, participants who had completed the second wave were invited to continue the study, and data on proactive behavior were collected. After invalid responses and unmatched questionnaires were removed, 514 valid matched responses were retained, yielding a valid response rate of 93.12%. After completing and submitting each survey wave, respondents received a cash incentive of RMB 2 through Credamo’s built-in reward system.

With respect to sample characteristics, 259 participants were men, accounting for 50.39% of the sample, and 255 were women, accounting for 49.61%, indicating a balanced gender distribution. In terms of age, 132 participants were aged 25 years or below, accounting for 25.68%; 195 were aged 26–35 years, accounting for 37.94%; 142 were aged 36–45 years, accounting for 27.63%; and 45 were aged over 45 years, accounting for 8.75%. With regard to educational attainment, 168 participants had an associate degree or below, accounting for 32.68%; 214 held a bachelor’s degree, accounting for 41.63%; 90 held a master’s degree, accounting for 17.51%; and 42 held a doctoral degree or above, accounting for 8.17%. In terms of organizational tenure, 148 participants had worked for under 1 year, accounting for 28.79%; 229 had worked for 1–3 years, accounting for 44.55%; 66 had worked for 4–6 years, accounting for 12.84%; and 71 had worked for over 6 years, accounting for 13.81%. The demographic profile of the participants is summarized in Table 1.

### 3.2. Measurement Tools

To ensure the reliability and validity of the survey instrument, all focal constructs in this study, including leader AI-focused attention, AI job role clarity, Affective rumination, Problem-solving pondering, and Proactive behavior, were measured with well established scales that have been widely used in leading international journals. In addition, to fully account for linguistic differences and employees’ levels of understanding, and to ensure the linguistic accuracy and cultural appropriateness of the original English scales in the Chinese context, all measures were translated into Chinese by following a rigorous translation and back translation procedure. This procedure facilitated participants’ comprehension of the questionnaire items. All core variables were measured on a five point Likert scale ranging from 1, strongly disagree, to 5, strongly agree. The specific items are presented in Appendix A.

Leader AI-focused attention. Leader AI-focused attention was measured using the five-item scale developed by Dong et al. (2025). The scale assesses employees’ perceptions of the extent to which their leaders devote attention to AI robots in the workplace, with AI robots understood as a concrete and observable carrier of AI technology. A sample item is, My leader always pays attention to what the AI robot is saying and doing. The Cronbach’s alpha coefficient for this scale was 0.871.

AI job role clarity. AI job role clarity was measured with the nine item scale developed by Chowdhury et al. (2022). A sample item is, I have clarity on how my roles and responsibilities will change as a result of AI adoption. The Cronbach’s alpha coefficient for this scale was 0.926.

Affective rumination. Affective rumination was measured with the five item scale developed by Querstret and Cropley (2012). A sample item is, Do you become tense when you think about work related issues during your free time? The Cronbach’s alpha coefficient for this scale was 0.932.

Problem-solving pondering. Problem-solving pondering was measured with the five item scale developed by Querstret and Cropley (2012). A sample item is, After work I tend to think of how I can improve my work related performance. The Cronbach’s alpha coefficient for this scale was 0.867.

Proactive behavior. Proactive behavior was measured with the nine item scale developed by Griffin et al. (2007). A representative item is, Makes suggestions to improve the overall efficiency of the organization. The Cronbach’s alpha coefficient for this scale was 0.941.

Control variables. Because participants’ demographic characteristics may influence the study results (Spector & Brannick, 2010), gender, age, educational level, and organizational tenure were included as control variables.

## 4. Results

### 4.1. Confirmatory Factor Analysis

Confirmatory factor analysis was conducted in Mplus 8.3. The five-factor model was specified as the baseline model, and four alternative models were further constructed, including a four-factor model in which problem-solving pondering and affective rumination were combined into one factor, a three-factor model in which AI job role clarity, problem-solving pondering, and affective rumination were combined into one factor, a two-factor model in which leader AI-focused attention, AI job role clarity, problem-solving pondering, and affective rumination were combined into one factor, and a one-factor model in which all variables were combined into a single factor. The results are presented in Table 2. The five-factor model shows a substantially better fit than the other four alternative models, with fit indices of χ^2^/df = 1.424, RMSEA = 0.029, CFI = 0.982, TLI = 0.980, and SRMR = 0.032, which indicates good discriminant validity among the five constructs measured in this study.

### 4.2. Reliability and Convergent Validity

Following the assessment of discriminant validity, the reliability and convergent validity of the study variables are further examined. As shown in Table 3, the standardized factor loadings of all measurement items on their corresponding latent constructs reach acceptable levels. In addition, the average variance extracted values of all variables exceed 0.50, indicating good convergent validity. The composite reliability values and Cronbach’s alpha coefficients are all above 0.70, suggesting satisfactory internal consistency reliability. Overall, the measures used in this study demonstrate good reliability and convergent validity.

### 4.3. Common Method Bias

Given that all survey measures were based on employee self-reports, common method bias may be a potential concern. Therefore, this study first conducted Harman’s single-factor test. The results showed that the first unrotated factor accounted for 27.18% of the total variance, which is below the commonly used threshold of 40%, suggesting that common method bias was unlikely to be a serious problem (Podsakoff et al., 2003). In addition, following the five-factor hypothesized model, this study added a latent common method factor to construct a six-factor model and compared it with the five-factor model. The results indicated that the inclusion of the common method factor did not substantially improve model fit, as the changes in fit indices were relatively small (ΔCFI = 0.015, ΔTLI = 0.015, ΔRMSEA = −0.011, ΔSRMR = −0.011). Taken together, these tests suggest that common method bias is unlikely to pose a serious threat to the findings of this study.

### 4.4. Descriptive Statistics and Correlation Analysis

Descriptive statistics and correlation analysis were conducted using SPSS 27.0, and the results are presented in Table 4. As shown in Table 4, leader AI-focused attention is positively correlated with affective rumination (r = 0.13, *p* < 0.01), problem-solving pondering (r = 0.47, *p* < 0.01), and proactive behavior (r = 0.35, *p* < 0.01). In addition, affective rumination is negatively correlated with proactive behavior (r = −0.32, *p* < 0.01), whereas problem-solving pondering is positively correlated with proactive behavior (r = 0.45, *p* < 0.01). These findings provide preliminary support for the hypothesized relationships among the main study variables.

### 4.5. Hypothesis Testing

Hierarchical regression analysis was conducted to examine the hypothesized relationships after controlling for demographic variables, including gender, age, educational level, and organizational tenure. The results are presented in Table 5. As shown in Model 2, leader AI-focused attention positively predicts problem-solving pondering (B = 0.46, *p* < 0.01), thus supporting H1a. Model 5 shows that leader AI-focused attention is also positively associated with affective rumination (B = 0.15, *p* < 0.01), thereby supporting H2a. In Model 8, leader AI-focused attention positively predicts proactive behavior (B = 0.38, *p* < 0.01).

Furthermore, when problem-solving pondering and affective rumination are simultaneously included in Model 9, problem-solving pondering is positively related to proactive behavior (B = 0.32, *p* < 0.01), whereas affective rumination is negatively related to proactive behavior (B = −0.31, *p* < 0.01). Meanwhile, the coefficient of leader AI-focused attention on proactive behavior decreases from 0.38 in Model 8 to 0.28 in Model 9, while remaining statistically significant at the 0.01 level. This pattern is consistent with the partial mediating roles of problem-solving pondering and affective rumination in the relationship between leader AI-focused attention and proactive behavior. Therefore, H1b, H1c, H2b, and H2c are supported.

To further examine the mediating roles of problem-solving pondering and affective rumination in the relationship between leader AI-focused attention and proactive behavior, the bootstrap method with 5000 resamples was used to estimate the 95% confidence intervals for the indirect associations. An indirect effect is considered significant when the confidence interval does not include zero. The results are reported in Table 6.

The results show that the indirect effect of leader AI-focused attention on proactive behavior through problem-solving pondering is 0.182, with a standard error of 0.031 and a 95% confidence interval of [0.125, 0.243], which does not include zero. This finding provides support for the mediating role of problem-solving pondering. At the same time, the indirect effect of leader AI-focused attention on proactive behavior through affective rumination is −0.054, with a standard error of 0.018 and a 95% confidence interval of [−0.091, −0.021], which also does not include zero. This result provides support for the mediating role of affective rumination. Accordingly, H1c and H2c receive further support. Taken together, these findings suggest that the relationship between leader AI-focused attention and proactive behavior exhibits a double-edged pattern. On the one hand, leader AI-focused attention is positively related to proactive behavior through increased problem-solving pondering. On the other hand, it is negatively related to proactive behavior through increased affective rumination.

With regard to the moderation tests, Model 3 in Table 5 shows that the interaction term between leader AI-focused attention and AI job role clarity is positively associated with problem-solving pondering (B = 0.24, *p* < 0.01), indicating that AI job role clarity positively moderates the relationship between leader AI-focused attention and problem-solving pondering. Therefore, H3 is supported. However, Model 6 shows that the interaction term between leader AI-focused attention and AI job role clarity is not significantly associated with affective rumination (B = −0.05, *p* > 0.05). Although the coefficient is in the hypothesized negative direction, the result does not provide sufficient statistical evidence that AI job role clarity weakens the positive association between leader AI-focused attention and affective rumination. Thus, H5 is not supported. This non-significant finding should not be interpreted as evidence for the opposite effect; rather, it suggests that the boundary role of AI job role clarity may be more evident in the cognitive pathway of problem-solving pondering than in the affective pathway of affective rumination. Accordingly, H6, which proposes that AI job role clarity negatively moderates the indirect association between leader AI-focused attention and proactive behavior through affective rumination, is also not supported.

**Figure 2:**
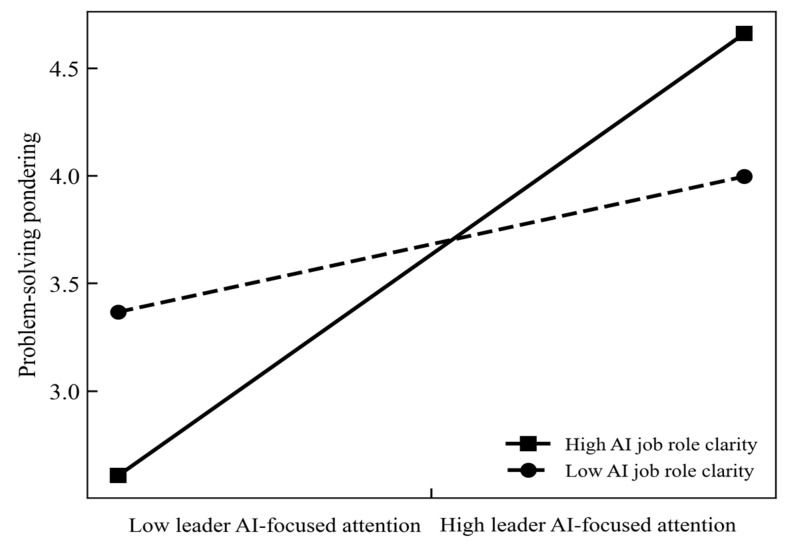
Moderating effect of AI job role clarity on problem-solving pondering.

To more clearly illustrate the moderating role of AI job role clarity in the relationship between leader AI-focused attention and problem-solving pondering, simple slope analysis was further conducted using values of AI job role clarity one standard deviation above and below the mean to represent high and low levels. As shown in Figure 2, when AI job role clarity is low, the positive association between leader AI-focused attention and problem-solving pondering is relatively weak. When AI job role clarity is high, this positive association becomes substantially stronger. These results suggest that the positive relationship between leader AI-focused attention and problem-solving pondering becomes stronger as AI job role clarity increases. In other words, AI job role clarity positively moderates the relationship between leader AI-focused attention and problem-solving pondering. Therefore, H3 receives further support.

**Figure 3:**
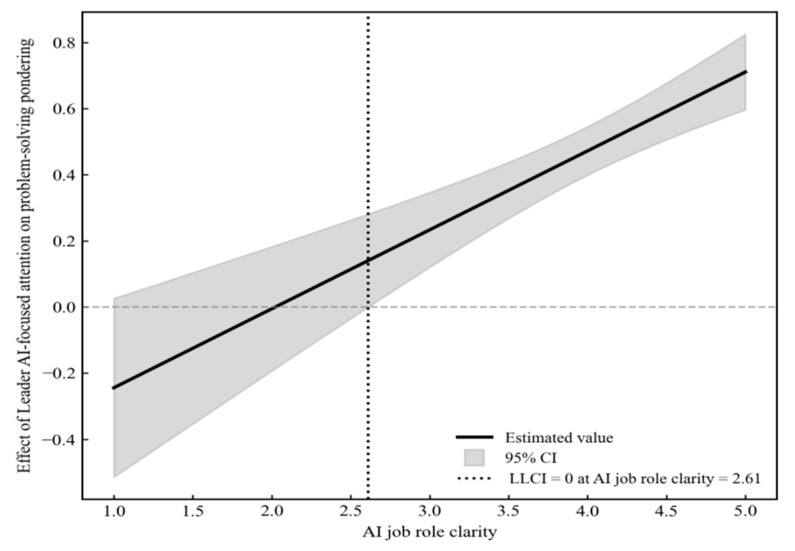
Johnson-Neyman analysis of the moderating effect of AI job role clarity.

In addition, the Johnson-Neyman technique was used to further examine this moderating effect, and the results are presented in Figure 3. The results indicate that the association between leader AI-focused attention and problem-solving pondering varies across different levels of AI job role clarity. Specifically, as AI job role clarity increases, the positive association between leader AI-focused attention and problem-solving pondering becomes stronger, which further suggests that AI job role clarity positively moderates this relationship.

To further examine the conditional indirect effect, bootstrap analysis was conducted to estimate the indirect effect of leader AI-focused attention on proactive behavior through problem-solving pondering at high and low levels of AI job role clarity, as well as the difference between these indirect effects. As shown in Table 7, when AI job role clarity is low, the indirect effect of leader AI-focused attention on proactive behavior through problem-solving pondering is 0.081, with a standard error of 0.035 and a 95% confidence interval of [0.019, 0.153], which does not include zero, indicating a significant indirect effect. When AI job role clarity is high, the indirect effect is 0.258, with a standard error of 0.037 and a 95% confidence interval of [0.189, 0.333], which also does not include zero, indicating that the indirect effect remains significant. In addition, the difference between the indirect effects under high and low levels of AI job role clarity is 0.178, with a standard error of 0.037 and a 95% confidence interval of [0.108, 0.251], which does not include zero, indicating a significant difference between the two indirect effects. Therefore, H4 is supported.

## 5. General Discussion

Drawing on work-related rumination theory, this study develops a moderated mediation model to examine the mechanism linking leader AI-focused attention to employee proactive behavior. Using a three-wave time-lagged design and empirical analysis of 514 valid responses, the results show that leader AI-focused attention is associated with employees’ problem-solving pondering and may also be accompanied by affective rumination, forming two distinct pathways related to employee proactive behavior. Specifically, leader AI-focused attention positively predicts both problem-solving pondering and affective rumination and is further linked to employee proactive behavior through these two forms of work-related rumination. In other words, problem-solving pondering and affective rumination serve as dual mediators in the relationship between leader AI-focused attention and employee proactive behavior.

Further, our findings suggest that the moderating role of AI job role clarity is primarily evident in the problem-solving pondering pathway. When employees have a clearer understanding of how AI is positioned in their jobs, where their responsibilities begin and end, and what AI-related collaboration requires, leader AI-focused attention is more likely to be translated into task-specific reflection on AI-related work and, in turn, to be positively linked to employee proactive behavior. By contrast, the moderating effect of AI job role clarity in the affective rumination pathway was not statistically significant. This pattern does not suggest that AI job role clarity is unimportant. Rather, it indicates that its role may lie mainly in shaping employees’ cognitive processing. Specifically, AI job role clarity may help employees interpret leaders’ AI-focused attention as a cue for task improvement, process optimization, and capability alignment, thereby facilitating more constructive problem-solving pondering. However, affective rumination may not diminish simply because employees have clearer role boundaries, as worries and uncertainty triggered by AI-related changes may persist even when role expectations are clarified. In this sense, the present study highlights the path-specific role of AI job role clarity in AI-related work contexts: it appears more relevant to the cognitive translation of leaders’ AI-focused attention than to the immediate reduction in negative psychological responses.

Regarding the unsupported H5 and H6, one possible explanation is that affective rumination in AI-related work contexts may not stem solely from ambiguous role boundaries. Compared with problem-solving pondering, affective rumination is more closely tied to emotion-laden repetitive thinking about threat, uncertainty, and anticipated loss. Such rumination may arise from AI anxiety, job insecurity, concerns about skill obsolescence, and perceived replacement threats. Even when employees clearly understand their current responsibilities in AI-related tasks, they may still worry that AI adoption will change performance evaluation criteria, reduce the value of their existing experience and skills, or increase uncertainty about their future career development. Thus, although AI job role clarity may help employees develop a more concrete understanding of current task requirements, it may not be sufficient to alleviate their emotional concerns about the longer-term implications of AI. Future research could further examine whether factors such as psychological safety, organizational support, leader reassurance, and employees’ trust in the organization’s AI implementation process serve as more relevant boundary conditions for the affective rumination pathway.

### 5.1. Theoretical Contributions

First, this study incorporates leader AI-focused attention into the explanatory framework of employee proactive behavior in AI-related work contexts, thereby enriching existing understanding of the factors associated with employees’ positive behavioral responses in such contexts. Prior research has begun to examine the relationship between AI-related contexts and employees’ positive behavioral responses, addressing issues such as employee voice, proactive behavior, career proactivity, and job crafting from the perspectives of organizational AI adoption, employee–AI collaboration, and proactive adaptation after AI implementation. For example, organizational AI adoption may be linked to employee voice through work engagement (Jeong, 2026); employee–AI collaboration may be associated with employee proactive behavior by reducing workload (Sun et al., 2025); and AI implementation may also be related to employees’ proactive job crafting (X. Lin et al., 2025). These studies provide an important foundation for understanding how employees proactively adapt to AI. Building on this line of research, the present study further introduces leader AI-focused attention as a contextual signal, suggesting that employee proactive behavior is related not only to AI technologies themselves and the process of using them, but also to the way leaders sustain attention to AI in daily management. In doing so, this study extends the explanation of the antecedents of employee proactive behavior in AI-related work contexts from the level of technology application and employee adaptation to the leadership context.

Second, by introducing work-related rumination theory, this study further explains how leader AI-focused attention is linked to employees’ psychological processing beyond the work context. Prior research on AI-related work contexts has shown that AI-related factors may correspond to psychological mechanisms in different directions. For example, leader AI awareness may be positively associated with employee voice through intrinsic motivation, while also being negatively associated with employee voice through job insecurity (Zhou & Lyu, 2025). Similarly, AI disruptive threat may be linked to employee innovative behavior through technology insecurity and thriving at work (Leong et al., 2025). These studies mainly reveal the coexistence of positive and negative mechanisms in AI-related work contexts. Building on this line of research, the present study extends attention to employees’ sustained psychological processing during nonwork time, showing that leader AI-focused attention is associated not only with task-oriented problem-solving pondering but also with affective rumination centered on technological pressure, role changes, and uncertainty. In this way, this study extends the discussion of the “double-edged” effects of AI-related work contexts from employees’ immediate responses at work to their post-work rumination processes. It also broadens, to some extent, the applicability of work-related rumination theory to organizational contexts shaped by artificial intelligence (Cropley & Zijlstra, 2011; Querstret & Cropley, 2012).

Furthermore, this study contributes to a deeper understanding of leadership-related contextual signals in human–AI interaction research. Existing studies on human–AI interaction have paid considerable attention to employees’ direct contact with AI technologies, AI use experiences, trust in AI, and adaptive behaviors toward AI, emphasizing how employees adjust their cognition, skills, and behaviors after AI enters the workplace (Glikson & Woolley, 2020; Raisch & Krakowski, 2021; Parker & Grote, 2022). For example, AI may not only replace certain tasks but also augment employees’ work capabilities and decision-making processes, meaning that employees’ responses in AI-related work contexts often involve complex psychological processes such as adaptation, reliance, vigilance, and role reorientation (Raisch & Krakowski, 2021; Tang et al., 2023). Compared with this line of research, the present study is likewise situated in AI-related work contexts, but its focus is not on how employees directly use AI. Instead, it examines how employees interpret the AI-focused attentional signals continuously conveyed by leaders. In this respect, this study, to some extent, complements the explanatory boundary of existing research on positive adaptation to AI. AI-related signals do not stem only from the technology itself or from AI-related communication among coworkers; they may also arise from leaders’ sustained attention to AI in daily management. Employees’ responses to such signals are not limited to whether they accept or use AI, but are also reflected in their continued thinking about work problems, role requirements, and future actions. This perspective connects the technology-adaptation view in human–AI interaction research with the process of leader attentional influence in organizational contexts.

Finally, this study offers a more specific account of the boundary role of AI job role clarity. Recent research on artificial intelligence and work has shown that technological change not only alters the way tasks are performed, but also reshapes job content, work boundaries, and the allocation of responsibilities (Cascio & Montealegre, 2016). At the same time, differences in employees’ behavioral responses in AI-related contexts have increasingly been examined through the lens of boundary conditions and contingency relationships (H. Lin et al., 2024; Sun et al., 2025). Building on this work, the present study further suggests that AI job role clarity reflects the extent to which employees clearly understand the functional boundaries of AI in their jobs, the division of responsibilities, and the modes of human–AI collaboration. It therefore represents a cognitive condition that is closely tied to AI-related work contexts. The findings indicate that AI job role clarity is mainly associated with a stronger relationship between leader AI-focused attention and problem-solving pondering, whereas it does not significantly moderate the affective rumination pathway. This finding does not weaken the theoretical value of AI job role clarity; rather, it reveals the path-specific nature of its role. AI job role clarity may be more likely to help employees understand AI-related tasks and collaboration requirements and to relate to more concrete problem-solving pondering. However, role clarity alone may not be sufficient to buffer worries, uncertainty, and emotion-laden repetitive thinking triggered by AI-related changes. Thus, this study provides a more cautious explanation of the boundary role of AI job role clarity and suggests that future research should further distinguish between cognitive and affective boundary conditions in AI-related work contexts.

### 5.2. Practical Implications

This study offers practical implications for how organizations can more effectively stimulate employee proactive behavior during AI-enabled transformation. As AI robots are increasingly incorporated into organizational work processes, leaders’ sustained attention to AI becomes an important cue through which employees interpret the organization’s technological orientation, task requirements, and future work standards. If leaders repeatedly emphasize only the importance of AI, efficiency improvement, or transformation pressure, employees may interpret such attention as performance pressure or job threat, thereby experiencing greater psychological burden. Therefore, organizations should guide managers at different levels to improve the way they communicate about AI and translate leader AI-focused attention into more concrete work improvement issues. In departmental meetings, project reviews, and performance conversations, leaders can place greater emphasis on how AI helps employees improve work quality, shorten process time, reduce repetitive tasks, and enhance their problem-solving capabilities, rather than merely stressing the need to adapt to AI as quickly as possible. For organizations, AI-related leadership communication can be incorporated into managerial training programs. Through case-based exercises, communication script design, and management scenario simulations, organizations can help managers develop a more supportive and development-oriented communication style.

In addition, organizations should pay attention to the development of AI job role clarity. The clearer employees are about the functional role of AI in their positions, the boundaries of their responsibilities, and the modes of human–AI collaboration, the more likely they are to interpret leader AI-focused attention as a positive signal related to task improvement and capability development. In practice, organizations can help employees clarify the specific role of AI robots in work processes, as well as employees’ own responsibilities in AI-related collaborative tasks, by updating job descriptions, providing guidelines for human–AI collaboration processes, establishing AI tool use protocols, clarifying task boundaries, and offering scenario-based training. These practices can not only reduce employees’ ambiguity regarding AI-related requirements but also help translate leader AI-focused attention into more concrete problem-solving pondering.

At the same time, managers should recognize that clarifying role boundaries does not mean that employees’ emotional strain will automatically disappear. AI job role clarity is more likely to help employees understand “what should be done” and “how to do it,” but it may not directly resolve the worries and uncertainty they experience in response to AI-related changes. Therefore, when promoting AI applications, organizations should not rely solely on process descriptions and responsibility allocation to alleviate employees’ concerns. They also need to attend to employees’ psychological experiences during AI use. Organizations can establish mechanisms for AI-related consultation, experience sharing, problem feedback, and peer support, so that employees can express their concerns about AI applications, job changes, and capability alignment in a timely manner. Managers should also provide timely feedback on employees’ attempts, improvements, and innovations in AI use, helping them recognize opportunities for growth in the new technological context. In this way, AI implementation is not merely an adjustment of technological tools and work processes; it can also become a process through which employees understand new tasks, develop new capabilities, and form employee proactive behavior.

### 5.3. Limitations and Future Research

First, although this study adopted a multi-wave survey design, which helps reduce potential bias associated with measuring all variables at a single point in time, the focal variables were still primarily based on employee self-reports, and employee proactive behavior was also self-rated. Therefore, the findings should be interpreted within the boundaries of self-reported survey data. Future research could incorporate supervisor ratings, coworker evaluations, or objective behavioral data to assess employee proactive behavior from multiple sources, thereby enhancing the robustness of the findings.

Second, although this study used screening questions to restrict the sample to technology-oriented firms and AI-robot-related work contexts, it did not further differentiate more fine-grained information such as industry sector, job function, and the intensity of AI implementation. This limitation may constrain the applicability of the findings across different AI-enabled work contexts. Specifically, industries may differ substantially in the maturity of AI applications, the depth of technological embeddedness, and the modes of human–AI collaboration. In organizations where AI applications are relatively mature, AI may have become deeply embedded in work processes, performance evaluation, and task coordination. In such contexts, employees may be more likely to interpret leader AI-focused attention in terms of task improvement, process optimization, and capability development. By contrast, in organizations where AI applications remain at the pilot or early implementation stage, employees may be more likely to interpret leader AI-focused attention as a signal of technological substitution, job adjustment, or performance pressure. Similarly, employees in different job roles may vary in the frequency and form of their contact with AI. Frontline operations, customer service, and production collaboration roles may involve more frequent direct interaction with AI systems, whereas back-office management, technical support, or functional roles may involve more indirect exposure to AI through data systems, process platforms, or decision-support tools. Therefore, the relationships between leader AI-focused attention, employees’ rumination processes, and employee proactive behavior may be jointly bounded by industry maturity, job characteristics, and the degree of AI embeddedness. Future research could collect more detailed information on industry type, job category, frequency of AI use, degree of AI embeddedness, and stage of AI implementation, and compare the mechanisms related to leader AI-focused attention across different AI-related work contexts. This would allow a more nuanced understanding of the boundary conditions of the proposed model.

Third, although the measurement of leader AI-focused attention in this study was based on an established scale, the scale was originally developed in an AI-robot work context. As the forms of AI applications in organizations continue to diversify, the AI systems employees encounter may include service robots, industrial robots, algorithmic management systems, intelligent decision-making systems, and generative AI tools. Future research could further examine the applicability of the leader AI-focused attention measure across different forms of AI technology and compare whether employees understand leader AI-focused attention differently across various AI application scenarios. Such efforts would further enhance the explanatory power of this construct across different organizational contexts.

Finally, in this study, AI job role clarity primarily strengthened the problem-solving pondering pathway, whereas its moderating effect on the affective rumination pathway was not statistically significant. This suggests that employees’ clear understanding of their AI-related work roles may be more likely to help them interpret task requirements, responsibility boundaries, and modes of collaboration, thereby fostering problem-solving pondering. However, worries, uncertainty, and emotion-laden repetitive thinking triggered by AI-related changes may require additional supportive conditions to be alleviated. Future research could further incorporate variables such as AI anxiety, career security, organizational support, psychological safety, and AI-related training support to examine which factors are more effective in reducing affective rumination in AI-related work contexts. Such efforts would further refine the explanation of the boundary conditions under which leader AI-focused attention is linked to employee proactive behavior.

## Figures and Tables

**Table 1:** Participant profile.

Variables	Category	Frequency	Percentage (%)
Gender	Male	259	50.39
	Female	255	49.61
Age	25 years or below	132	25.68
	26–35 years	195	37.94
	36–45 years	142	27.63
	Over 45 years	45	8.75
Education	Associate degree or below	168	32.68
	Bachelor’s degree	214	41.63
	Master’s degree	90	17.51
	Doctoral degree or above	42	8.17
Tenure	Under 1 year	148	28.79
	1–3 years	229	44.55
	4–6 years	66	12.84
	Over 6 years	71	13.81
Total		514	100

**Table 2:** Results of confirmatory factor analysis.

Model	χ^2^/df	RMSEA	CFI	TLI	SRMR
Five-factor model: LAA, AJRC, PSP, AR, PB	1.424	0.029	0.982	0.980	0.032
Four-factor model: LAA, AJRC, PSP + AR, PB	4.102	0.078	0.864	0.853	0.127
Three-factor model: LAA, AJRC + PSP + AR, PB	7.494	0.112	0.714	0.693	0.156
Two-factor model: LAA + AJRC + PSP + AR, PB	9.934	0.132	0.604	0.577	0.183
One-factor model: All variables combined	14.757	0.164	0.389	0.349	0.207

Note: LAA = Leader AI-focused attention; AJRC = AI job role clarity; PSP = problem-solving pondering; AR = affective rumination; PB = proactive behavior.

**Table 3:** Results of reliability and convergent validity.

Variables	Items	Standardized Factor Loadings	AVE	CR	Cronbach’s α
Leader Al-focused attention	LAA1	0.853	0.661	0.907	0.871
	LAA2	0.784			
	LAA3	0.797			
	LAA4	0.813			
	LAA5	0.817			
Affective rumination	AR1	0.899	0.787	0.949	0.932
	AR2	0.879			
	AR3	0.89			
	AR4	0.89			
	AR5	0.879			
Problem-solving pondering	PSP1	0.826	0.656	0.905	0.867
	PSP2	0.827			
	PSP3	0.772			
	PSP4	0.8			
	PSP5	0.823			
Proactive behavior	PB1	0.879	0.683	0.951	0.941
	PB2	0.853			
	PB3	0.856			
	PB4	0.809			
	PB5	0.813			
	PB6	0.733			
	PB7	0.823			
	PB8	0.816			
	PB9	0.848			
AI job role clarity	AJRC1	0.826	0.631	0.939	0.926
	AJRC2	0.815			
	AJRC3	0.793			
	AJRC4	0.777			
	AJRC5	0.762			
	AJRC6	0.774			
	AJRC7	0.805			
	AJRC8	0.778			
	AJRC9	0.817			

**Table 4:** Means, standard deviations, and correlation coefficients of variables.

Variables	M	SD	1	2	3	4	5	6	7	8
1. Gender	0.50	0.50								
2. Age	2.19	0.92	0.04							
3. Education	2.01	0.91	0.04	0.01						
4. Tenure	2.12	0.98	0.03	0.44 **	−0.04					
5. Leader AI-focused attention	3.73	1.05	0.02	0.03	0.02	0.01				
6. Affective rumination	2.90	1.19	−0.22 **	0.03	−0.16 **	0.06	0.13 **			
7. Problem-solving pondering	3.69	1.04	−0.01	0.07	0.10 *	−0.02	0.47 **	−0.11 *		
8. AI job role clarity	3.80	0.94	0.41 **	0.04	0.05	0.05	0.13 **	−0.45 **	0.01	
9. Proactive behavior	3.63	1.16	0.04	0.01	0.14 **	−0.06	0.35 **	−0.32 **	0.45 **	0.07

Note: * *p* < 0.05 ** *p* < 0.01.

**Table 5:** Regression analysis results.

Variables	Problem-Solving Pondering			Affective Rumination			Proactive Behavior		
	M1	M2	M3	M4	M5	M6	M7	M8	M9
Constant	3.39 **	1.72 **	1.88 **	3.37 **	2.83 **	4.50 **	3.33 **	1.96 **	2.30 **
Gender	−0.04	−0.05	0.01	−0.52 **	−0.53 **	−0.08	0.09	0.07	−0.07
Age	0.11	0.09	0.07	0.02	0.01	0.02	0.05	0.04	0.01
Education	0.11 *	0.10 *	0.10 *	−0.20 **	−0.20 **	−0.18 **	0.17 **	0.16 **	0.06
Tenure	−0.07	−0.06	−0.04	0.07	0.07	0.08	−0.09	−0.09	−0.05
Leader Al-focused attention		0.46 **	0.42 **		0.15 **	0.22 **		0.38 **	0.28 **
Problem-solving pondering									0.32 **
Affective rumination									−0.31 **
AI job role clarity			−0.03			−0.59 **			
Leader Al-focused attention × AI job role clarity			0.24 **			−0.05			
R^2^	0.02	0.24	0.28	0.08	0.09	0.27	0.02	0.14	0.33
F	2.34	31.98	28.56	10.59	10.62	26.40	3.24	17.21	35.57

Note: * *p* < 0.05 ** *p* < 0.01.

**Table 6:** Mediation effect Test results.

Path	Effect	Boot SE	BootLLCI	BootULCI
Leader Al-focused attention → Problem-solving pondering → Proactive behavior	0.182	0.031	0.125	0.243
Leader Al-focused attention → Affective rumination → Proactive behavior	−0.054	0.018	−0.091	−0.021

**Table 7:** Moderated mediation effect test.

Level of AI Job Role Clarity	Effect	Boot SE	Boot LLCI	Boot ULCI
Low (−1 SD)	0.081	0.035	0.019	0.153
High (+1 SD)	0.258	0.037	0.189	0.333
Difference	0.178	0.037	0.108	0.251

## Data Availability

The original contributions presented in this study are included in the article. Further inquiries can be directed to the corresponding author.

## Appendix A

The following are the survey items.

Leader Al-focused attention:

1. My leader always pays attention to the appearance of the Al robot.
2. My leader always pays attention to external physical conditions related to the Al robot (such as obstacles in movement paths).
3. My leader always pays attention to the activity status of the Al robot (such as electricity).
4. My leader always pays attention to the impact of the Al robot on others, such as Customers.
5. My leader always pays attention to what the Al robot is saying and doing.

Problem-solving pondering:

1. I find solutions to work-related problems in my free time.
2. I think about how I can improve my work performance during my free time.
3. I reflect on work-related problems in order to solve them.
4. I think about alternative ways to deal with work-related issues.
5. I think about how to complete work-related tasks more effectively.

Affective rumination:

1. Do you become tense when you think about work-related issues in your free time?
2. Do work-related issues trouble you after work?
3. Do you feel tired when you think about work-related issues in your free time?
4. Do you become irritated when you think about work-related issues during your free time?
5. Do you feel annoyed when you think about work-related issues after work?

AI job role clarity:

1. I have clarity on how my roles and responsibilities will change as a result of AI adoption.
2. I have clarity on the expectations from my work as a result of AI adoption.
3. I have clarity on the organisational strategy towards AI adoption.
4. I have clarity on how my performance will be measured in an environment where AI is used.
5. I have clarity how the nature of my work will change.
6. I have clarity on how AI systems will be used in my organisation.
7. I have clarity on why AI systems will be used for specific tasks in my organisation.
8. I have clarity on the role of human intelligence within a collaborative working environment.
9. I have clarity in social hierarchy where AI and human will co-exist (i.e., social status of employees being higher than AI systems) will drive AI adoption within my organisation.

Proactive behavior:

1. I proactively look for better ways to perform my core work tasks.
2. I come up with ideas to improve how my core tasks are carried out.
3. I make changes to improve the way I complete my core tasks.
4. I suggest ways to make my work unit more effective.
5. I develop new and better methods to help my work unit perform better.
6. I improve the way my work unit gets work done.
7. I make suggestions to improve the overall effectiveness of the organization.
8. I involve myself in changes that help improve the overall effectiveness of the organization.
9. I come up with ways to increase efficiency within the organization.
